# Reporter cell lines to screen for inhibitors or regulators of the KRAS-RAF-MEK1/2-ERK1/2 pathway

**DOI:** 10.1042/BCJ20240015

**Published:** 2024-03-07

**Authors:** Laura Weatherdon, Kate Stuart, Megan Cassidy, Alberto Moreno de la Gándara, Hanneke Okkenhaug, Markus Muellener, Grahame Mckenzie, Simon J. Cook, Rebecca Gilley

**Affiliations:** 1 Signalling Programme, The Babraham Institute, Babraham Research Campus, Cambridge CB22 3AT, U.K.; 2 Phoremost, Unit 7, The Works, Unity Campus, Pampisford, Cambridge CB22 3FT, U.K.; 3 Imaging Facility, The Babraham Institute, Babraham Research Campus, Cambridge CB22 3AT, U.K.

**Keywords:** BRAF, ERK1/2, gene reporters, KRAS, MEK1/2, small molecules

## Abstract

The RAS-regulated RAF–MEK1/2–ERK1/2 signalling pathway is activated in cancer due to mutations in RAS proteins (especially KRAS), BRAF, CRAF, MEK1 and MEK2. Whilst inhibitors of KRAS^G12C^ (lung adenocarcinoma) and BRAF and MEK1/2 (melanoma and colorectal cancer) are clinically approved, acquired resistance remains a problem. Consequently, the search for new inhibitors (especially of RAS proteins), new inhibitor modalities and regulators of this pathway, which may be new drug targets, continues and increasingly involves cell-based screens with small molecules or genetic screens such as RNAi, CRISPR or protein interference. Here we describe cell lines that exhibit doxycycline-dependent expression KRAS^G12V^ or BRAF^V600E^ and harbour a stably integrated EGR1:EmGFP reporter gene that can be detected by flow cytometry, high-content microscopy or immunoblotting. KRAS^G12V^ or BRAF^V600E^-driven EmGFP expression is inhibited by MEK1/2 or ERK1/2 inhibitors (MEKi and ERKi). BRAFi inhibit BRAF^V600E^-driven EmGFP expression but enhance the response to KRAS^G12V^, recapitulating paradoxical activation of wild type RAF proteins. In addition to small molecules, expression of iDab6, encoding a RAS-specific antibody fragment inhibited KRAS^G12V^- but not BRAF^V600E^-driven EmGFP expression. Finally, substitution of EmGFP for a bacterial nitroreductase gene allowed KRAS^G12V^ or BRAF^V600E^ to drive cell death in the presence of a pro-drug, which may allow selection of pathway inhibitors that promote survival. These cell lines should prove useful for cell-based screens to identify new regulators of KRAS- or BRAF-dependent ERK1/2 signalling (drug target discovery) as well as screening or triaging ‘hits’ from drug discovery screens.

## Introduction

The RAS-regulated RAF–MEK1/2–ERK1/2 signalling pathway links growth factor-activated receptors to changes in gene expression in the nucleus, thereby controlling whether cells progress through the G1-to-S-phase cell cycle transition or arrest and differentiate [1]. Receptor-driven activation of the RAS GTPases allows them to recruit the RAF protein kinases (ARAF, BRAF or CRAF) to the plasma membrane where they are activated as dimer pairs [2,3]. Active RAF dimers phosphorylate serine residues in the activation loop of MEK1 and MEK2 (p-MEK1/2), resulting in their activation. Active p-MEK1/2 in turn activates ERK1/2 by phosphorylating threonine and tyrosine residues in the T-E-Y motif in the ERK1/2 activation loop (p-ERK1/2) [4]. This promotes release of activated p-ERK1/2 from MEK1/2, enabling p-ERK1/2 to bind to and phosphorylate cytoplasmic substrates, such as the kinase RSK, or enter the nucleus to phosphorylate transcription factors such as ETS and AP-1 proteins to regulate gene expression [1,5].

The RAS-regulated ERK1/2 signalling pathway is frequently activated in cancer [6] and developmental syndromes (RASopathies) [7,8] due to activating mutations in a variety of pathway components including the RAS proteins (especially KRAS), BRAF, CRAF, MEK1 and MEK2. These mutations lead to inappropriate activation of ERK1/2 which disrupts key developmental decisions and/or drives the acquisition of cancer hallmarks. This has driven drug discovery efforts to identify inhibitors of this pathway. BRAF and MEK inhibitors (BRAFi and MEKi) are now approved for the treatment of BRAF^V600E/K^ melanoma [9,10] and colorectal cancer [11]; the MEKi, Selumetinib, is approved for the treatment of paediatric neurofibromatosis, a RASopathy driven by deregulated RAS activation [12] and ERK1/2 inhibitors are in development [13]. Although RAS proteins were long viewed as ‘undruggable’, significant recent progress has seen the development of KRAS inhibitors that specifically target the KRAS^G12C^ mutant allele which have now been approved for the treatment of KRAS^G12C^-mutant lung adenocarcinoma [14].

Despite these remarkable advances, acquired resistance to these targeted agents remains a problem and the search for new inhibitors (especially of RAS proteins), new inhibitor modalities and new regulators, which represent new drug targets, continues. As well as traditional *in vitro* enzyme (or enzyme cascade) screens, this increasingly involves cell-based screens with small molecules or RNAi-, CRISPR- or protein or peptide-based libraries; these may allow identification of short-lived but druggable protein conformations that are not faithfully reproduced with purified recombinant proteins. Indeed, U0126, one of the earliest MEKi, was discovered in a cell-based gene reporter screen [15].

Here we describe cell lines that exhibit doxycycline-dependent expression of KRAS^G12V^ or BRAF^V600E^ and harbour a stably integrated EGR1:EmGFP reporter gene, the product of which can be detected by flow cytometry, high-content microscopy or western immunoblotting. We show that these cell lines faithfully recapitulate the distinct sensitivities of KRAS^G12V^ or BRAF^V600E^ to anti-RAS antibodies, RAFi, MEKi or ERKi. We also adapt an established gene-directed enzyme prodrug therapy (GDEPT) strategy so that the EGR1 promoter and 5′UTR drives expression of a bacterial nitroreductase gene allowing KRAS^G12V^ or BRAF^V600E^ to drive prodrug-dependent cell death; this may allow selection, by survival, and identification of novel pathway regulators. These cell lines should prove useful for cell-based screens to identify new regulators of KRAS-dependent ERK1/2 signalling (drug target discovery) as well as triaging ‘hits’ from more traditional drug discovery screens.

## Results

### Generation and validation of HeLa TetR KRAS^G12V^ or HeLa TetR BRAF^V600E^ EGR1:EmGFP reporter cell lines

**Figure 1.**
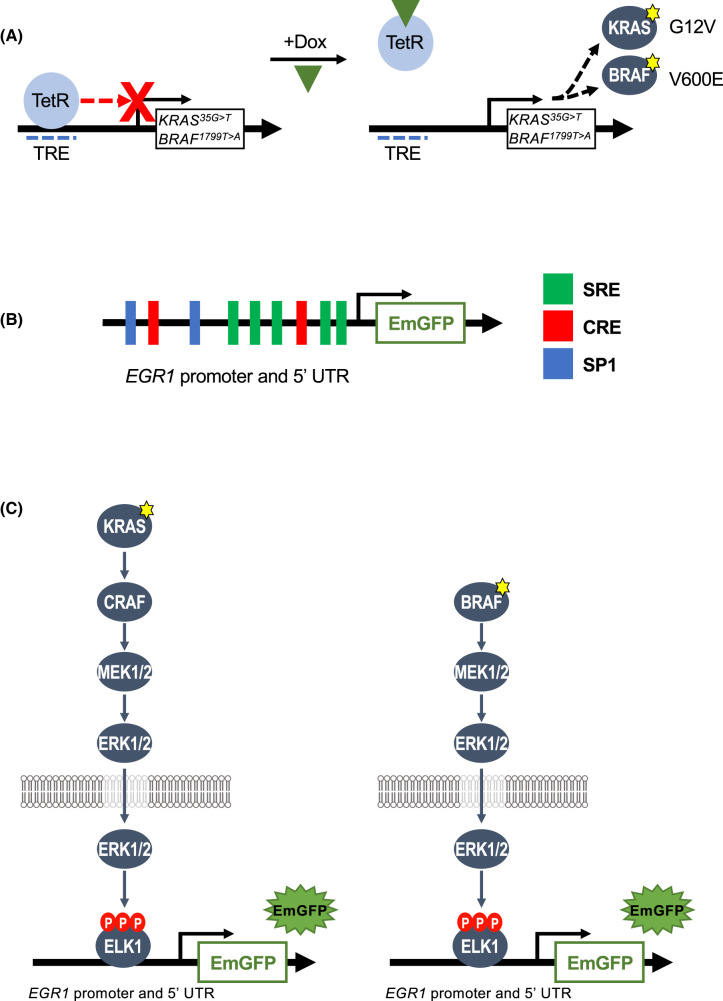
Components of KRAS^G12V^- or BRAF^V600E^-driven ERK1/2-dependent EmGFP reporter. (**A**) Schematic of the doxycycline-inducible Tet Repressor (TetR) system used in this study. HeLa cells were engineered to express either *KRAS^35G>T^* (encoding KRAS^G12V^) or *BRAF^1799T>A^* (encoding BRAF^V600E^) under the control of tetracycline response element (TRE). In the absence of doxycycline (Dox) TetR binds the TRE and inhibits transcription. Binding of Dox to the Tet causes it to dissociate from the TRE, allowing transcription of *KRAS^35G>T^* or *BRAF^1799T>A^*. (**B**) The promoter region and 5′UTR of EGR1 (−778 to +280), inserted upstream of the EmGFP cDNA. The promoter consists of five critical ERK1/2 responsive serum-response elements (SREs), as well as two cAMP-responsive elements (CREs) and two SP1 elements. (**C**) HeLa TetR KRAS^G12V^ cells (left) or HeLa TetR BRAF^V600E^ cells (right) have been engineered to express an EGR1:EmGFP reporter construct. Treatment of these clones with Dox should drive expression of KRAS^G12V^ or BRAF^V600E^, leading to activation and nuclear entry of ERK1/2 which then phosphorylates and activates ELK1 which in turns drives transcription of EmGFP from the EGR1 promoter and 5′UTR.

**Figure 2.**
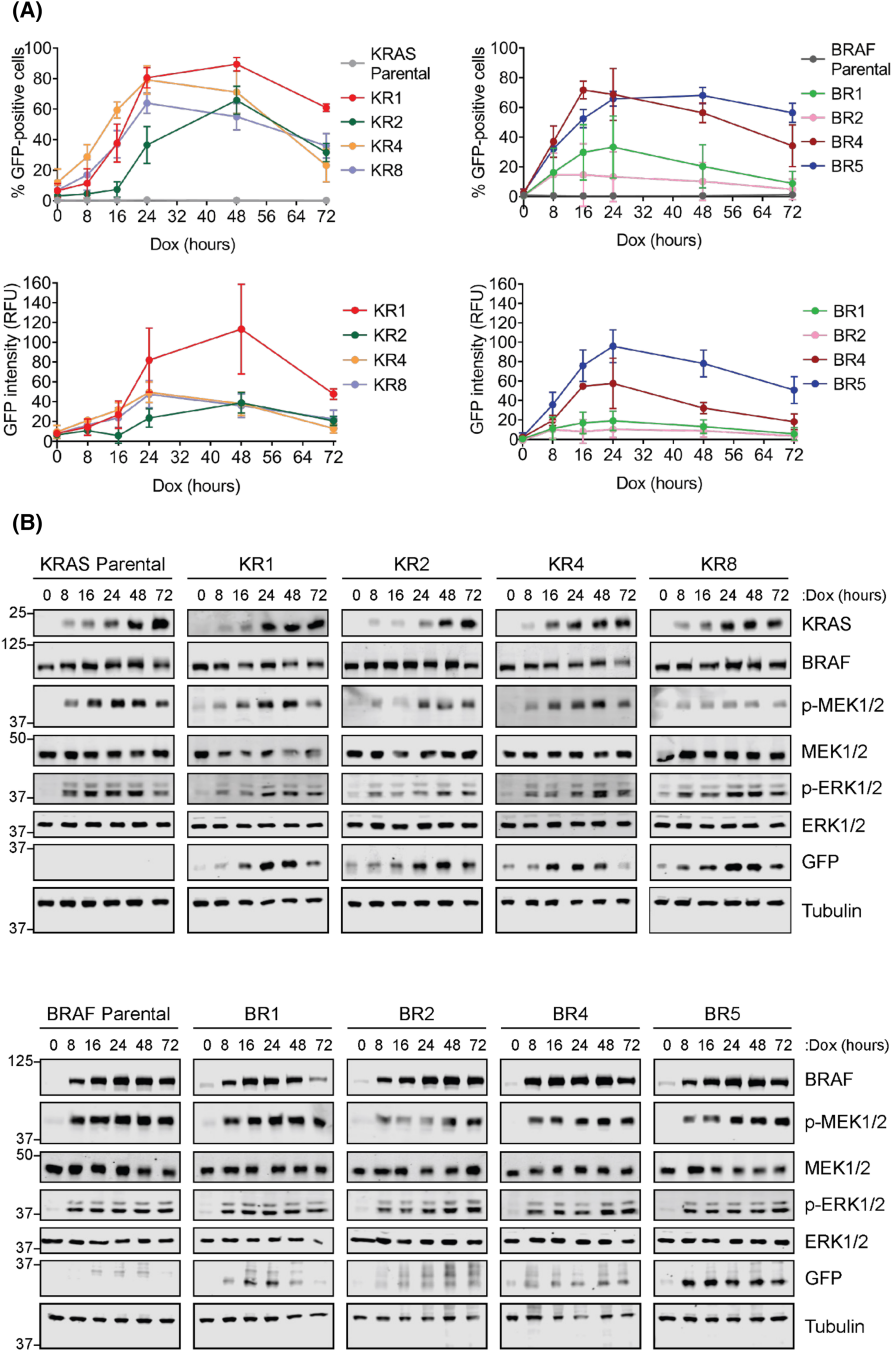
Doxycycline treatment drives a time-dependent increase in EmGFP expression in HeLa KRAS^G12V^ and BRAF^V600E^ clones expressing an EGR1:EmGFP reporter. (**A**) Parental HeLa TetR KRAS^G12V^ cells (left) or HeLa TetR BRAF^V600E^ cells (right) along with clones expressing an EGR1:EmGFP reporter (KR1, KR2, KR4, KR8; BR1, BR2, BR4, BR5) were treated with 100 ng/ml Doxycycline (Dox) for between 0 and 72 h. Cells were fixed, stained with DAPI and imaged by high content microscopy. Results were analysed to determine the percentage of GFP-positive cells and mean GFP intensity, with GFP intensity from parental cells taken as background. Results represent the mean ± S.D. from three independent experiments, each performed in triplicate. (**B**) The cells described above (**A**) were treated as above and whole cell lysates were fractionated by SDS–PAGE and probed with the indicated antibodies. Results are representative of three independent experiments.

To generate cells lines with conditional expression of KRAS^G12V^ or BRAF^V600E^ we employed the Tet-repressor (TetR) system in which TetR binds the tetracycline response element (TRE) and inhibits transcription [16]. When cells are treated with Doxycycline (Dox), Dox binds the TetR and causes it to dissociate from the TRE, allowing Dox-dependent transcription of the gene of interest (cDNAs encoding KRAS^G12V^ or BRAF^V600E^) (Figure 1A). HeLa cell lines with Dox-dependent expression of KRAS^G12D^ were previously described [17] and we used the same system to generate HeLa cell lines with Dox-dependent expression of KRAS^G12V^ or BRAF^V600E^ (see Figure 2). To monitor ERK1/2 signalling output in high-throughput we elected to use a gene reporter system. The immediate-early gene *EGR1* is a well-characterised ERK1/2 gene target [1]. The *EGR1* promoter contains five serum response elements (SREs) (Figure 1B) which serve as binding sites for SRF:ELK transcription factor complexes; the ELK component is phosphorylated and activated by ERK1/2 [1,18]. In this way activation of the ERK1/2 pathway drives *EGR1* transcription. In preliminary experiments we cloned the Emerald green fluorescent protein (EmGFP) reporter gene downstream of the *EGR1* promoter and 5′UTR and prepared a series of deletion mutants (Supplementary Figure S1A) to identify the construct that gave maximal KRAS^G12V^-driven ERK1/2-dependent EmGFP expression. All constructs exhibited Dox-inducible expression of EmGFP that was strongly inhibited by the MEKi, PD184352 (Supplementary Figure S1B); the largest, full length (FL) *EGR1* promoter and 5′UTR construct comprising −778 to +280 bp (numbering relative to transcriptional start) was found to give maximal expression (Supplementary Figure S1B). Notably, all constructs exhibited a low basal expression of EmGFP that was absent from untransfected cells; this modest basal activity probably reflects basal ERK1/2 activation driven by growth factors in foetal bovine serum (FBS). The FL *EGR1* promoter and 5′UTR was taken forward for further analysis with the goal of generating cells in which inducible expression of KRAS^G12V^ or BRAF^V600E^ resulted in EmGFP expression from the EGR1:EmGFP reporter (Figure 1C). The use of these cell lines could offer a method for identifying novel regulators of the RAS–RAF–MEK1/2–ERK1/2 pathway, as detecting changes in EmGFP expression is an easy, robust and quantitative way of assessing signalling output and perturbations. Furthermore, comparing EmGFP expression driven by mutant KRAS^G12V^ with that driven by BRAF^V600E^, might identify proteins that regulate the pathway at the level of RAS.

HeLa TetR KRAS^G12V^ or HeLa TetR BRAF^V600E^ cell lines were transfected with the EGR1:EmGFP reporter, clonal cell lines were isolated and screened for Dox-inducible expression of EmGFP. The parental cell lines (HeLa TetR KRAS^G12V^ or HeLa TetR BRAF^V600E^) and four clones of each (KR1, 2, 4 and 8 or BR1, 2, 4 and 5) were taken forward for further analysis (Figure 2A,B). To confirm that expression of these oncogenes promoted expression of EmGFP, cells were treated with Dox for various time points between 0 and 72 h and analysed by high content microscopy (Figure 2A). For all the clones expressing the EGR1:EmGFP reporter, treatment with Dox increased both the percentage of GFP-positive cells and the mean GFP intensity in a time-dependent manner. The peak expression of EmGFP was earlier, at 16–24 h, in cells expressing BRAF^V600E^ compared with the peak at 24–48 h in cells expressing KRAS^G12V^. The magnitude of the response varied greatly between the clones but in all cases EmGFP expression was consistently lower after 72 h of Dox treatment (Figure 2A). To confirm that EmGFP expression in these clones correlated with signalling through the ERK1/2 pathway, cell extracts were analysed by western blot. Consistent with the high content microscopy data, BRAF^V600E^ expression increased more rapidly than KRAS^G12V^ expression, BRAF^V600E^ being strongly expressed at 8 h in all BR clones (Figure 2B). This difference in kinetics of KRAS^G12V^ and BRAF^V600E^ expression was reflected in downstream signalling; in virtually all BRAF clones the increase in p-MEK1/2 and p-ERK1/2 levels was maximal after only 8 h of Dox treatment (Figure 2B) whereas p-MEK1/2 and p-ERK1/2 increased more gradually in the KR clones. In both systems, the delay between KRAS/BRAF expression and EmGFP response was expected due to the requirement for *de novo* synthesis of both KRAS^G12V^ or BRAF^V600E^ and EmGFP expression. EmGFP levels were more variable in clones with inducible BRAF^V600E^, with only the BR5 clone showing a strong induction of EmGFP expression by both western blot and high content microscopy. Parental KR and BR clones showed no inducible expression of EmGFP.

**Figure 3.**
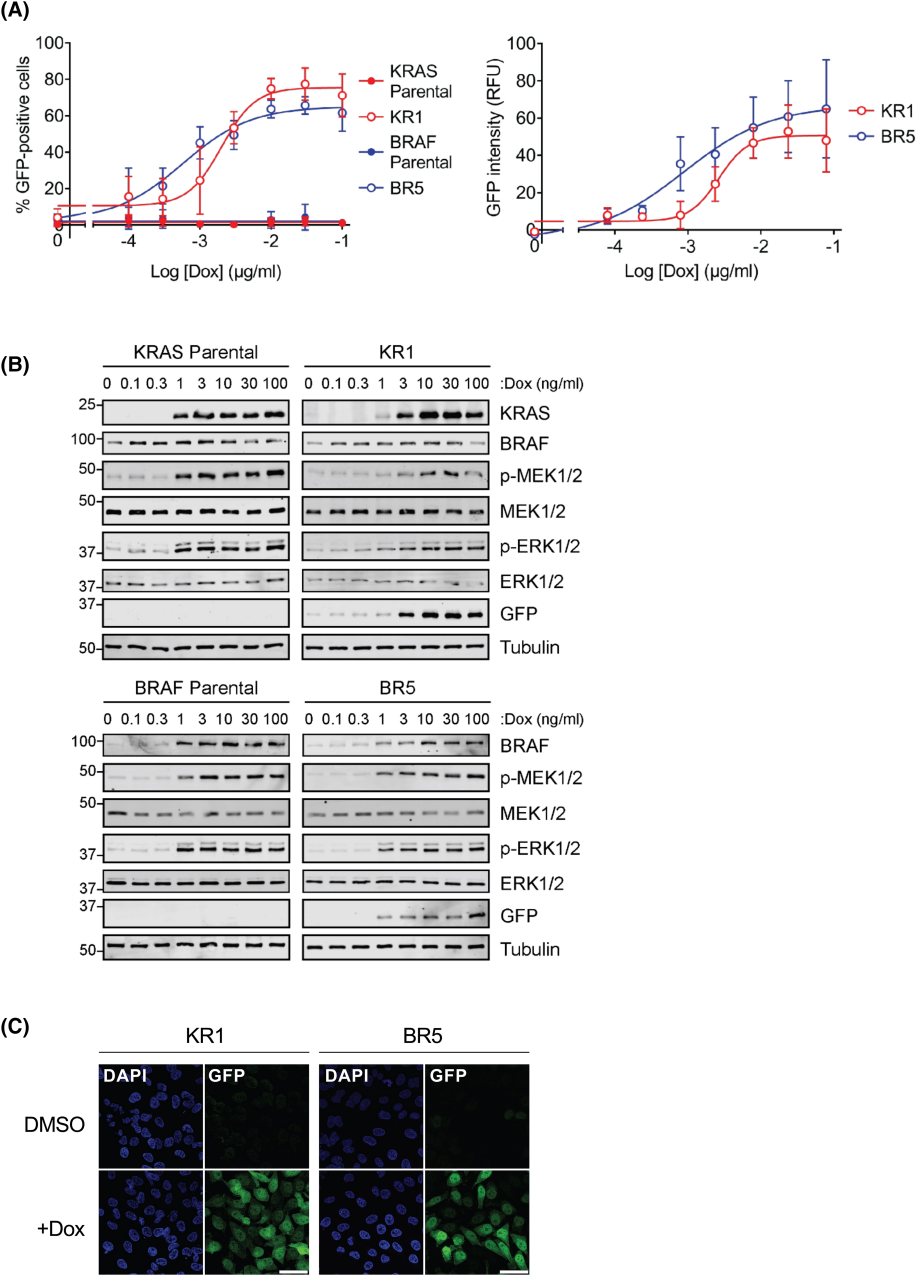
Doxycycline drives a dose-dependent increase in EmGFP expression in HeLa KR1 and BR5 clones. (**A**) HeLa clones expressing an ERK1/2-responsive emGFP reporter construct, with either doxycycline-inducible KRAS^G12V^ (KR1) or BRAF^V600E^ (BR5), were treated with a range of Doxycycline (Dox) doses up to 100 ng/ml for 24 h. Parental cells were also treated as a control. Cells were fixed, stained with DAPI and analysed by high content microscopy. Results were analysed to determine the percentage of GFP-positive cells and mean GFP intensity. GFP intensity from parental cells was taken as background. Results represent the means ± S.D. from four independent experiments, each performed in triplicate. (**B**) Cells were treated as above (**A**) were fractionated by SDS–PAGE and probed with the indicated antibodies by western blotting. Results are representative of three independent experiments. (**C**) Representative images of clones KR1 and BR5 described in (**A**). Cells were treated with either DMSO or 100 ng/ml Doxycycline (Dox) for 24 h. Cells were then fixed and mounted on slides with Vectashield mounting media containing DAPI. Cells were imaged on LEICA Stellaris 8 confocal microscope. Results are representative of three independent experiments. Scale bars represent 50 µm.

Based on these findings the KRAS KR1 and BRAF BR5 clones were chosen as the most suitable for further experiments as they exhibited a clear, maximal increase in EmGFP expression after 24 h of Dox induction. To determine the dose of Dox that drives maximal EmGFP expression in both clones, cells were treated with a range of Dox concentrations, alongside the parental cell lines. A dose of 10–100 ng/ml was able to produce the greatest EmGFP expression in both clones (Figure 3A). In both clones, western blot analysis confirmed that the dose-dependent increase in EmGFP protein expression correlated with a dose-dependent Dox-inducible increase in KRAS or BRAF expression, p-MEK1/2 and p-ERK1/2 (Figure 3B). Representative images of KR1 or BR5 cells treated with or without Dox to activate ERK1/2 signalling and EmGFP expression are shown in Figure 3C.

### MEK1/2 and ERK1/2 inhibitors abolish EmGFP expression induced by KRAS^G12V^ or BRAF^V600E^

**Figure 4.**
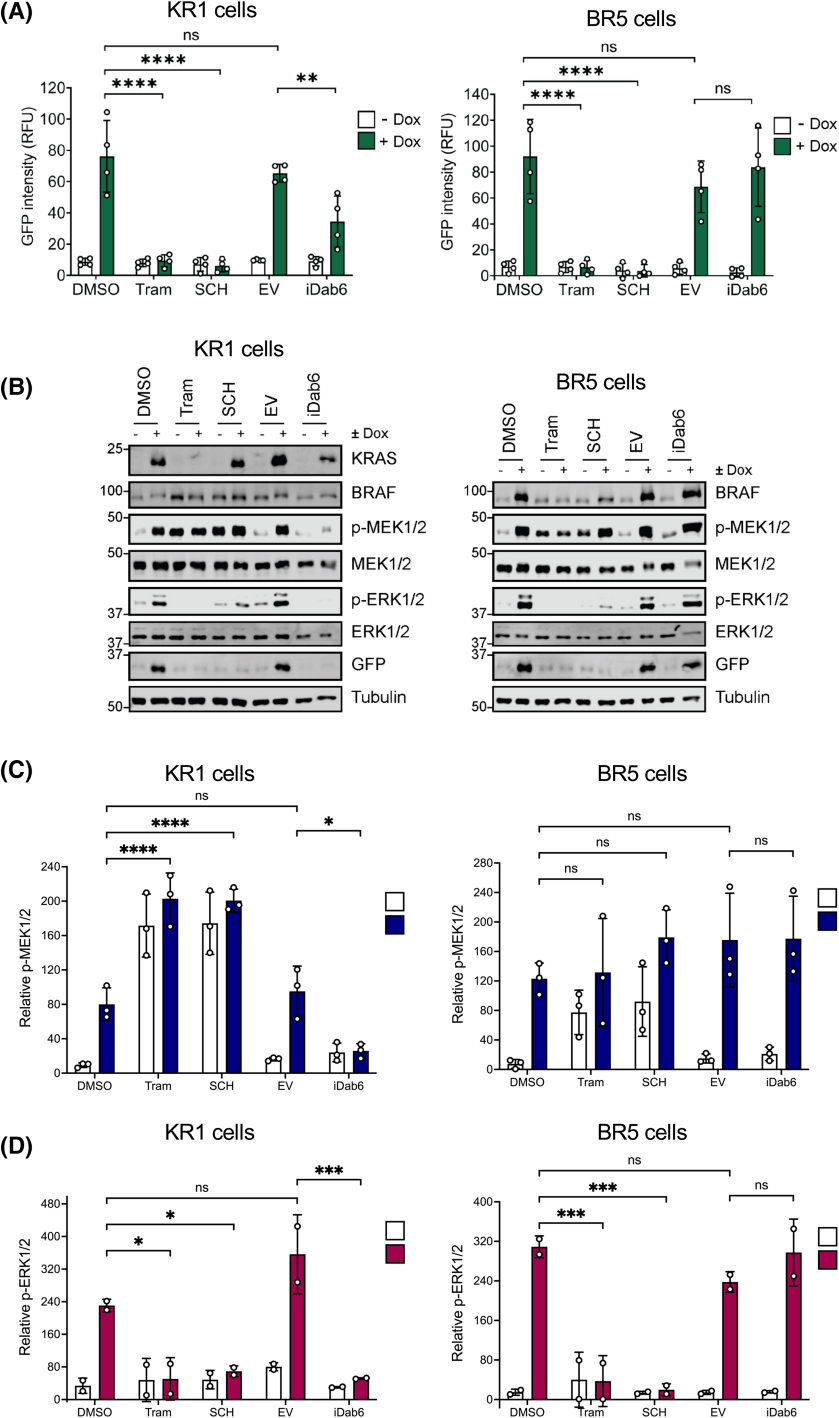
Inhibiting the RAS–RAF–MEK1/2–ERK1/2 pathway blocks induction of EmGFP in KR1 and BR5 clones. (**A**) Inhibitors to the RAS–RAF–MEK1/2–ERK1/2 pathway were tested for their effects on EmGFP expression in HeLa EGR1:EmGFP reporter cell lines expressing doxycycline-inducible KRAS^G12V^ (KR1, left) or BRAF^V600E^ (BR5, right). Cells were transfected with iDab6 or empty (EV) as a control and incubated for 4 h, before media was replaced with fresh culture medium. At the same time, appropriate cells were treated with either 100 nM Trametinib (Tram) or 100 nM SCH772984 (SCH). All cells were then treated with 100 ng ml Dox or DMSO for 24 h before fixation. Cells were stained with DAPI and imaged with a high content microscope. Results were analysed to determine the mean GFP intensity within the population; GFP intensity from parental cells was taken as background. Results represent the means ± SD from four independent experiments, each performed in triplicate. (**B**) Cells were treated as above, and whole cell lysates fractionated by SDS–PAGE and blotted with the indicated antibodies. Results are representative of three independent experiments. (**C** and **D**) Quantification of western blot analysis for (**C**) p-MEK1/2 relative to MEK1/2 or (**D**) p-ERK1/2 relative to ERK1/2. Results are the mean normalised blot quantification ± SD from at least two independent experiments. *P*-values: ^ns^*P *> 0.05, *P < 0.05, **P < 0.01, ***P < 0.001, ****P < 0.0001 analysed by two-way ANOVA followed by Tukey's multiple comparison test.

To confirm that KRAS^G12V^- or BRAF^V600E^-dependent expression of EmGFP was dependent on signalling through the RAS–RAF–MEK–ERK1/2 pathway we used the potent and selective ERK1/2 inhibitor, SCH772984, and the potent, allosteric MEK1/2 inhibitor, Trametinib, which is clinically approved for the treatment of melanoma. Both SCH772984 and Trametinib completely abolished Dox-inducible EmGFP expression in the KR1 and BR5 clones, confirming that activation of the EGR1:EmGFP reporter was absolutely dependent on ERK1/2 signalling (Figure 4A,B). Despite inhibiting EmGFP expression (Figure 4A,B) and ERK1/2 phosphorylation (Figure 4D), both inhibitors led to a significant increase in p-MEK1/2, that was especially apparent in the KR1 clone (Figure 4B,C). This can be explained by a loss of ERK1/2-dependent inhibitory phosphorylation of BRAF and CRAF, the kinases that usually act as RAS-dependent dimers to drive the phosphorylation of MEK1/2. In cells with wild type RAF proteins ERK1/2 directly phosphorylate BRAF and CRAF to inhibit them, preventing MEK1/2 phosphorylation as part of a homeostatic feedback loop [19,20]. Inhibition of MEK1/2 or ERK1/2 prevents this feedback inhibition, resulting in increased RAF activity and MEK1/2 phosphorylation. This is much less apparent in cells expressing BRAF^V600E^ because this mutant signals as a monomer and is refractory to ERK1/2-dependent feedback inhibition [21]. Thus, the HeLa TetR KRAS^G12V^ or HeLa TetR BRAF^V600E^ cell lines faithfully reproduced a key difference between KRAS^G12V^ or BRAF^V600E^ signalling.

### Idab6, a RAS-specific antibody fragment inhibits EmGFP expression induced by KRAS^G12V^ but not BRAF^V600E^

To further assess the selectivity of the HeLa TetR KRAS^G12V^ or HeLa TetR BRAF^V600E^ cell lines we used iDab6 a single domain antibody fragment that binds RAS proteins with high specificity and inhibits downstream signalling [22]. Transient transfection of an iDab6 expression plasmid into the KR1 clone caused a significant reduction in EmGFP expression and phosphorylation of MEK1/2 and ERK1/2 (Figure 4A–D). Critically, iDab6 had no effect on signalling or EmGFP expression induced by BRAF^V600E^ which is downstream and independent of RAS, again showing that these cell lines reproduced a key difference between KRAS^G12V^ or BRAF^V600E^ signalling. In the course of these experiments we noted that treatment with MEK or ERK inhibitors caused a decrease in expression of inducible KRAS^G12V^ or BRAF^V600E^ proteins; this effect was variable between experiments but suggests that there may be an ERK1/2-responsive element in the Tet-regulated gene expression system. However, this did not affect the interpretation of our results. For example, Trametinib inhibited KRAS expression whereas SCH772984 did not, yet they both abolished ERK1/2 phosphorylation and EmGFP expression (Figure 4B).

### RAF inhibitors enhance KRAS^G12V^-driven EmGFP expression but inhibit the response to BRAF^V600E^

**Figure 5.**
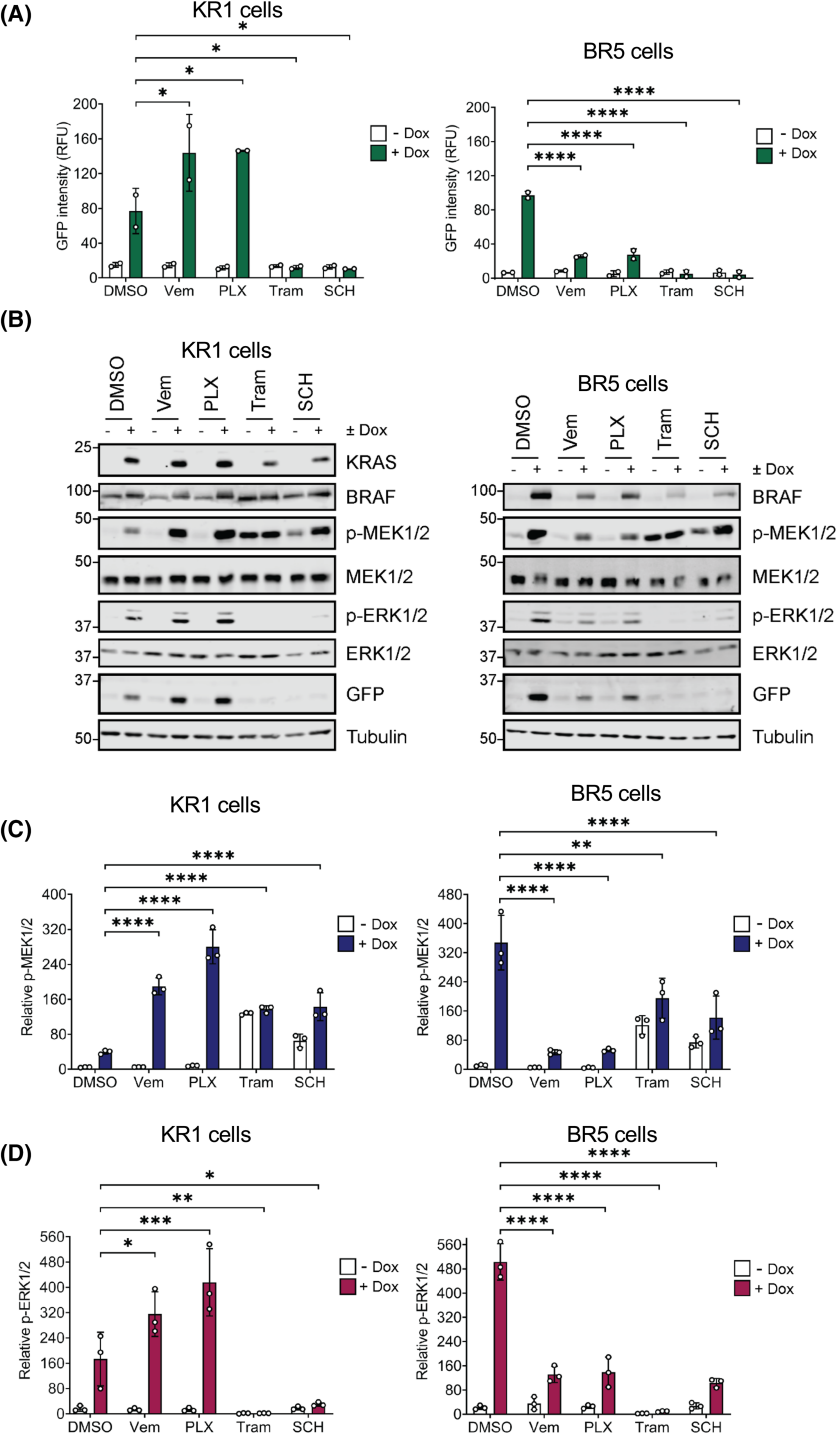
BRAF^V600E^ inhibitors drive paradoxical activation of ERK1/2 signalling and EmGFP expression in KR1 clone. (**A**) HeLa cells containing an ERK1/2-responsive EmGFP reporter construct and expressing doxycycline-inducible KRAS^G12V^ (KR1, left) or BRAF^V600E^ (BR5, right) were treated with 1 µM Vemurafenib (Vem), 1 µM PLX4720 (PLX), 100 nM Trametinib (Tram), or 100 nM SCH772984 (SCH). Cells were then treated with 100 ng/ml Doxycycline (Dox), or DMSO as a control, and incubated for 24 h. Cells were fixed and stained with DAPI before being imaged on a high content microscope. Results were analysed to determine the mean GFP intensity within the population; GFP intensity from parental cells was taken as background. Results represent the means ± SD from two independent experiments. (**B**) Cells were treated as above, and whole cell lysates fractionated by SDS–PAGE and blotted with the indicated antibodies. Results are representative of three independent experiments. (**C and D**) Quantification of western blot analysis for (**C**) p-MEK1/2 relative to MEK1/2 or (**D**) p-ERK1/2 relative to ERK1/2. Results are the mean normalised blot quantification ± S.D. from three independent experiments. *P*-values: ^ns^*P *> 0.05, *P < 0.05, **P < 0.01, ***P < 0.001, ****P < 0.0001 analysed by two-way ANOVA followed by Tukey's multiple comparison test.

We next tested the effects of Vemurafenib and PLX4720, both BRAF^V600E^-selective inhibitors [9]. We predicted these inhibitors would reduce ERK1/2 pathway activation and EmGFP expression driven by BRAF^V600E^, while increasing signalling driven by KRAS^G12V^ due to their ability to drive paradoxical activation of wild type RAF dimers [23–25]. This turned out to be the case; both BRAF inhibitors enhanced KRAS^G12V^-driven p-MEK1/2, p-ERK1/2 and EmGFP expression in the KR1 clone (Figure 5). In contrast these inhibitors caused a strong reduction in BRAF^V600E^-driven p-MEK1/2, p-ERK1/2 and EmGFP expression in the BR5 clone (Figure 5).

### Inhibitors of PI3K, p38 MAPK or JNK do not inhibit KRAS^G12V^- or BRAF^V600E^-dependent ERK1/2 signalling or EmGFP expression

**Figure 6.**
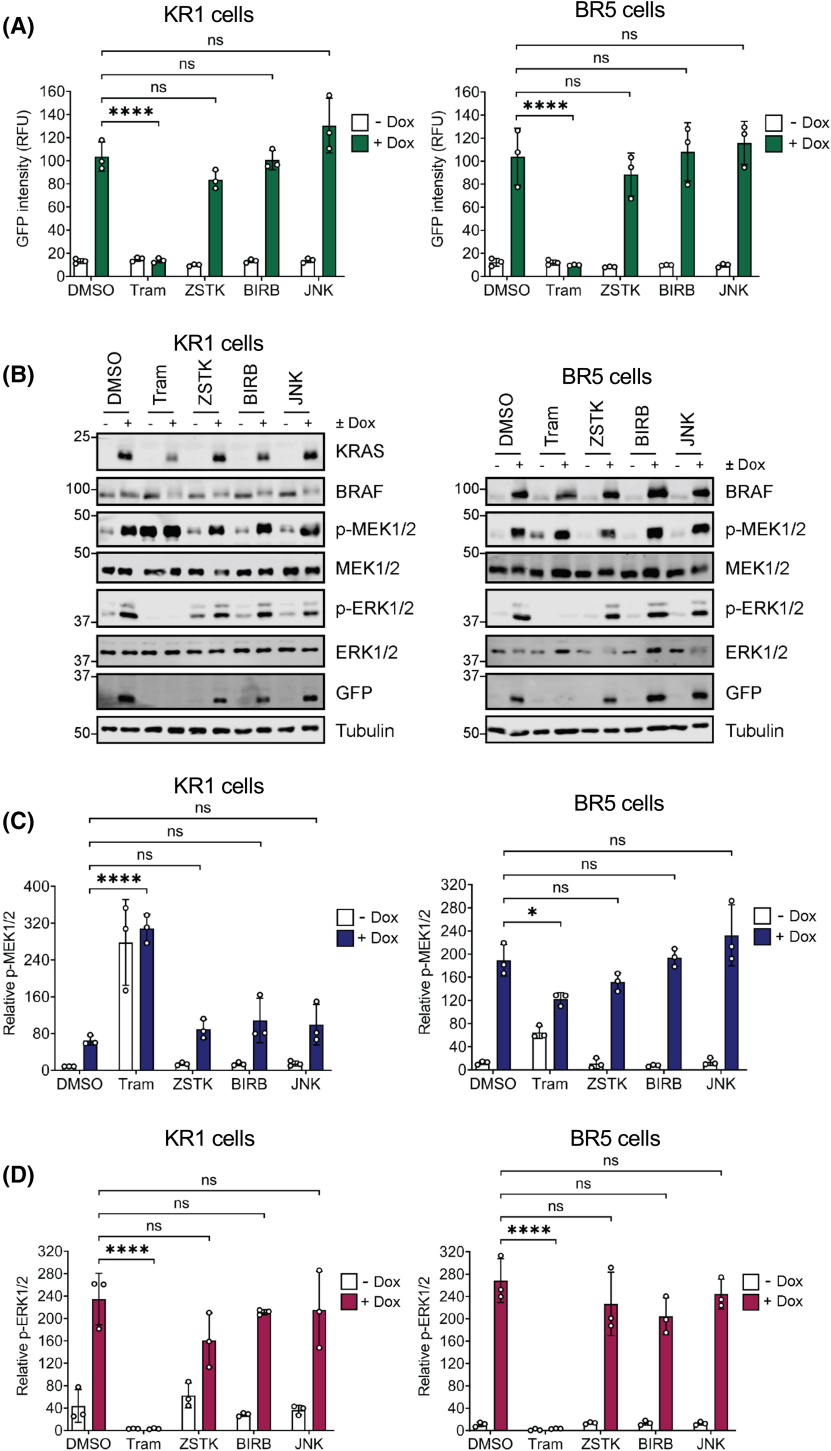
Inhibiting PI3K, JNK or p38MAPK does not inhibit KRAS^G12V^ or BRAF^V600E^ -inducible EGR1:EmGFP activation. (**A**) HeLa cells containing the EGR1:EmGFP reporter construct and expressing Doxycycline-inducible KRAS^G12V^ (KR1) or BRAF^V600E^ (BR5) were treated with 100 nM Trametinib (Tram), 200 nM ZSTK474 (ZSTK), 50 nM BIRB796 (BIRB), or 5 µM JNK inhibitor VIII (JNK). Cells were then treated with 100 ng/ml Doxycycline (Dox) or DMSO for 24 h, fixed and stained with DAPI before being imaged on a high content microscope. Results were analysed to determine the mean GFP intensity within the population. GFP intensity from parental cells was taken as background. Results represent the means ± SD from three independent experiments. (**B**) Cells were treated as above, and whole cell lysates fractionated by SDS–PAGE and blotted with the indicated antibodies. Results are representative of three independent experiments. (**C** and **D**) Quantitative western blot analysis of blots shown in figure (**B**). (**C**) p-MEK1/2 relative to MEK1/2 or (**D**) p-ERK1/2 relative to ERK1/2. Results are the mean normalised blot quantification ± S.D. from three independent experiments. *P*-values: ^ns^*P *> 0.05, *P < 0.05, **P < 0.01, ***P < 0.001, ****P < 0.0001 analysed by two-way ANOVA followed by Tukey's multiple comparison test.

In addition to the RAF–MEK1/2–ERK1/2 cascade, RAS can directly activate multiple other effector pathways including the PI3K pathway [2,26]. Furthermore, during prolonged RAS activation (as is used here) direct RAS effector pathways can ‘feed-forward’ to activate other signalling pathways including the JNK and p38MAPK pathways, which regulate some of the same processes as ERK1/2 [27]. To confirm that EGR1:EmGFP expression was solely driven by ERK1/2 and not these other pathways we used the pan-PI3K inhibitor ZSTK474, JNK inhibitor VIII and the p38 inhibitor, BIRB796. As a positive control, Trametinib again abolished EmGFP expression and ERK1/2 signalling in both KR1 and BR5 clones whereas ZSTK474, JNK inhibitor VIII and BIRB796 had no significant effect (Figure 6A–D). Trametinib again increased p-MEK1/2 in the KR1 clones whilst abolishing p-ERK1/2.

To confirm that these inhibitors were effective, KR1 and BR5 cells were treated with the protein synthesis inhibitor Anisomycin, which drives activation of JNK and p38MAPK, leading to phosphorylation of downstream targets, JUN and MAPKAP Kinase-2 (MK2) respectively. Phosphorylation of JUN was blocked by treatment with JNK inhibitor VIII as indicated by increased mobility through SDS–PAGE gels, and by a reduction in JUN abundance (JNK phosphorylation of JUN autoregulates its expression) (Supplementary Figure S2A). Inhibition of p38 MAPK by BIRB796 was confirmed by loss of p-MK2 signal and by increased mobility through SDS–PAGE gels (Supplementary Figure S2A). Finally, the efficacy of the PI3K inhibitor ZSTK474 was confirmed by a strong reduction in p-S473 AKT in both KR1 and BR5 cells (Supplementary Figure S2B). Thus, effective doses of PI3K, JNK and p38 MAPK inhibitors had no effect on KRAS^G12V^- or BRAF^V600E^-dependent ERK1/2 signalling or EmGFP expression.

### Transforming ERK1/2 signalling into a death signal by expression of a bacterial nitroreductase

**Figure 7.**
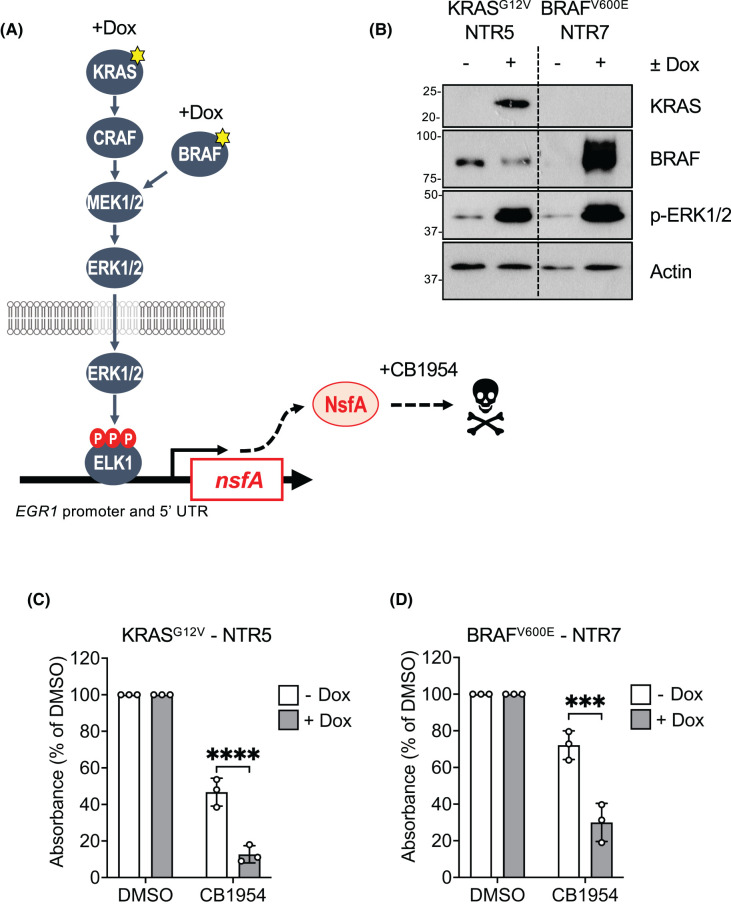
Transforming ERK1/2 signalling into a death signal by expression of the bacterial nitroreductase gene *nsfA*. (**A**) Schematic showing the Tet inducible nitroreducatse system. HeLa TetR KRAS^G12V^ or HeLa TetR BRAF^V600E^ cells have been engineered to express an EGR1:NsfA reporter construct. Treatment of these clones with Dox drives expression of KRAS^G12V^ or BRAF^V600E^ leading to ERK1/2-dependent expression of the NfsA nitroreductase enzyme. NsfA can catalyse the conversion of the CB1954 prodrug into cytotoxic DNA cross-linking intermediates, driving cell death. (**B**) Cells were treated with 100 ng/ml Doxycycline (Dox) or DMSO for 48 h, and whole cell lysates fractionated by SDS–PAGE and blotted with the indicated antibodies. (**C**) Cells were treated as above in the presence or absence of 200 μM CB1954 and fixed in methanol/acetic acid (3:1 w/v) for 10 min followed by staining with a 0.1% crystal violet solution. Cells were then solubilised in 10% acetic acid and the absorbance was read at 590 nm in a plate reader. Results were analysed to determine the absorbance relative to the DMSO counterpart. Results represent the means ± SD from three independent experiments *P*-values: ^ns^*P *> 0.05, *P < 0.05, **P < 0.01, ***P < 0.001, ****P < 0.0001 analysed by two-way ANOVA followed by Tukey's multiple comparison test.

The preceding results highlighted the utility of the HeLa TetR KRAS^G12V^ EGR1:EmGFP (KR1) and HeLa TetR BRAF^V600E^ EGR1:EmGFP (BR5) cell lines for reporting on inhibition of the ERK1/2 pathway by small molecules or by expression of proteins. However, screens that rely on inhibition of signalling or gene expression as their readout can yield a high number of ‘false positives’ as they can identify molecules that have off-target effects or are simply cytotoxic. An alternative and increasingly preferred approach is to use ‘survival screens’ in which the inducing stimulus drives cell death and small molecules, CRISPR guides or genetically-encoded peptides are selected by their ability to promote survival. RAS-dependent ERK1/2 signalling promotes survival by reducing the expression or activity of pro-death BCL2 proteins such as BIM and BMF and promoting the expression of prosurvival proteins such as MCL1 [28]. We sought to adapt our system so that KRAS^G12V^- or BRAF^V600E^-dependent ERK1/2 signalling promoted cell death. The *nfsA* and *nfsB* genes of *Escherichia coli* encode oxygeninsensitive nitroreductase (Ntr) enzymes, dimeric flavoproteins that catalyse the reduction in nitroaromatics and quinones by NADPH; this reduction is required for the activity of some antibiotics [29]. Ntr enzymes have attracted interest in GDEPT in cancer [30,31]. The NfsA Ntr enzyme can activate the pro-drug CB1954 to generate DNA cross-linking intermediates that drive apoptosis so we replaced the EmGFP cDNA in the EGR1:EmGFP construct with a cDNA encoding NsfA so that activation of ERK1/2 would drive NsfA expression and sensitise cells to CB1954 (Figure 7A).

HeLa TetR KRAS^G12V^ or HeLa TetR BRAF^V600E^ cells were transduced with a lentiviral construct containing the EGR1:NsfA reporter and stable clones selected and screened for Dox-driven, CB1954-dependent cell death. Candidate KRAS^G12V^ or BRAF^V600E^-inducible clones (NTR5, NTR7 respectively) were identified and we confirmed that these cells retained Dox-inducible expression of KRAS^G12V^ or BRAF^V600E^ and activation of ERK1/2 (Figure 7B). Notably, both NTR5 and NTR7 cells exhibited a reduction in viability when treated with CB1954 in the absence of Dox; this was more pronounced in the NTR5 cells (Figure 7C,D). This likely represents basal activity of the EGR1 5′UTR promoter construct described above (Supplementary Figure S1B) since these cells were maintained in low levels of FBS that contain growth factors that activate ERK1/2 signalling. Regardless, pre-treatment with Dox followed by addition of CB1954 resulted in a significant reduction in viability in both clones; in both cases this was a consistent 40–45% reduction in cell viability when treated with Dox and CB1954 (Figure 7C,D). Thus, KRAS^G12V^- or BRAF^V600E^-driven ERK1/2 activation was transformed into a cell death signal by use of the EGR1:NsfA and CB1954 GDEPT pair.

## Discussion

The clinical success of small molecule inhibitors of BRAF, MEK1/2 and most recently KRAS^G12C^ is limited by innate and acquired drug resistance which frequently arises through reactivation of ERK1/2. This validates the development of ERK1/2 inhibitors [13] but also highlights the need for new approaches to inhibit this pathway, new inhibitor modalities and the identification of additional regulators of the pathway that may be non-intuitive drug targets. This increasingly involves cell-based screens with libraries of small molecules or RNAi-, CRISPR- or protein interference-based libraries. One of the advantages of cell-based screens is that they may allow identification of short-lived but druggable protein conformations that are not always faithfully reproduced in purified recombinant proteins or *in vitro* enzyme kinase cascade assays. This study describes the generation and validation of HeLa TetR KRAS^G12V^ EGR1:EmGFP and HeLa TetR BRAF^V600E^ EGR1:EmGFP reporter cell lines that can be used to monitor inhibition of the ERK1/2 pathway by small molecules (RAFi, MEKi, ERKi) or by genetically encoded peptides or proteins (e.g. iDab6).

These ERK1/2-dependent reporter cell lines exhibit many desirable properties for screening. The use of the EmGFP reporter system allows rapid, high-throughput, semi-quantitative analysis of signal pathway output using high-content imaging platforms. This system can easily be adapted for flow cytometry or FACS to sort cells with the highest or lowest ERK1/2 signal output; in this way cells with ERK1/2 pathway inhibition could be identified and sequenced to identify the CRISPR guide, RNAi or protein interference peptides driving such changes (drug target discovery). The Dox-inducible system allows tight conditional control of pathway activation kinetics allowing choice of the optimal signal over background (Figure 2). Whilst the magnitude of the response varied between the clones, the kinetics were very similar. Notably, in all cases EmGFP expression consistently declined from 48 to 72 h of Dox treatment; this likely reflects the ERK1/2-dependent expression of negative feedback modulators such as the DUSP family of ERK1/2 phosphatases [32]. The TetR system also provides the ability to drive distinct thresholds of ERK1/2 signalling by varying Dox dose (Figure 3); this point is important since the magnitude or stoichiometry of ERK1/2 activation can direct distinct cell fates such as cell proliferation or cell cycle arrest [33,34]. Another advantage of these reporter cells is that they allow activation of the ERK1/2 pathway at the level of RAS or RAF. Hits in a KRAS-driven screen could be rapidly counter-screened using the BRAF-driven system; hits in both systems might be novel BRAF, MEK1/2 or ERK1/2 inhibitors, or might target regulators of the pathway such as scaffold proteins. Such ‘on pathway’ hits could be easily confirmed by screening for p-ERK1/2 or p-RSK in multiplexed high-content mode [35]. Finally, the system could easily be expanded to allow Dox-inducible expression of oncogenic mutants of SHP2, HRAS, NRAS, MEK1 or MEK2.

A key necessity in such ERK1/2 reporter systems is that they faithfully reproduce sensitivity to established RAS–RAF–MEK1/2–ERK1/2 pathway inhibitors. As expected, both ERKi (SCH772984) and MEKi (Trametinib) inhibited EmGFP expression whether it was driven by KRAS^G12V^ or BRAF^V600E^ (Figures 4 and 5). In contrast, BRAF^V600E^-selective inhibitors blocked BRAF^V600E^-driven EmGFP expression but actually enhanced KRAS^G12V^-driven ERK1/2 activation and EmGFP expression (Figure 5). This reflects a fundamental difference between wild type RAF proteins, which signal as RAS-dependent dimers, and BRAF^V600E^ which is constitutively active and signals as a monomer. BRAF-selective inhibitors completely inhibit BRAF^V600E^, thereby inhibiting MEK1/2 and ERK1/2 activation. In contrast, wild type RAF proteins signal as RAS-dependent dimers and RAFi promote RAS-dependent dimerisation and paradoxical activation of wild-type RAF proteins [23–25]. These contrasting responses to RAFi were clearly captured in our KRAS^G12V^- and BRAF^V600E^-driven reporter cell lines. Differential sensitivity to iDab6, a single domain antibody fragment that binds and inhibits RAS proteins, was also apparent as this construct inhibited KRAS^G12V^- but not BRAF^V600E^-driven EmGFP expression. This also demonstrates that our system can be used for both small molecules (drug discovery) and genetically-encoded peptide screens (drug target discovery). Finally, signalling diverges at the level of RAS proteins since they can activate multiple effector pathways including direct activation of PI3K signalling as well as indirect activation of JNK and p38MAPK, likely through feedforward signalling. Critically, inhibitors of PI3K, JNK or p38MAPK had no effect on KRAS^G12V^- or BRAF^V600E^-driven EmGFP expression (Figure 6), again underlying the selectivity of these reporter cell lines.

Cell-based screens that rely on inhibition of signalling pathways or gene expression as their readout can yield ‘false positives’ as they can identify molecules that are simply cytotoxic. For these reasons ‘survival screens’ are increasingly favoured as an alternative and involve the inducing stimulus driving cell death with small molecules, CRISPR guides or genetically-encoded peptides selected by their ability to promote survival. We initially sought to transform KRAS signalling into a death stimulus by expressing the pro-apoptotic protein BIM from the EGR1 promoter and 5′UTR; however, the low level of basal expression from this system (Supplementary Figure S1) translated into a 60–80% basal cell death even in the absence of Dox-dependent KRAS^G12V^ or BRAF^V600E^ expression (data not shown), likely reflecting the potent pro-death effects of BIM [28]. As an alternative we deployed an established GDEPT strategy in which EGR1-dependent NsfA expression was activated by KRAS^G12V^ or BRAF^V600E^ before treating cells with the pro-drug CB1954. This strategy did not abolish, but reduced, basal cell death, providing a consistent window of KRAS^G12V^- or BRAF^V600E^-driven, CB1954-dependent cell death. This approach requires further optimisation in terms of varying CB1954 and Dox dose and treatment times and the use of FBS replacement medium to limit basal ERK1/2 signalling and NsfA expression. Alternatively, since Trametinib treatment abolished basal ERK1/2 phosphorylation but left basal GFP intensity unaffected in several experiments the basal cell death may reflect input from other pathways. JNK, p38 and PI3K pathway inhibition was without effect but another candidate that should be considered is input from PKA signalling since the EGR1 5′UTR contains at least two cAMP response elements (CREs). Unfortunately, PKA inhibitors exhibit too many off-target effects to assess this directly and future work should include mutating the CREs in the EGR1 5′UTR to see if this reduces basal EmGFP expression and basal cell death. Regardless, it demonstrates that this strategy shows promise for screening for small molecules, proteins or peptides that inhibit KRAS^G12V^- or BRAF^V600E^-dependent ERK1/2 signalling with cell survival as the readout.

In summary, we demonstrate the validation of HeLa TetR KRAS^G12V^ EGR1:EmGFP and HeLa TetR BRAF^V600E^ EGR1:EmGFP reporter cell lines that can be used to monitor inhibition of the ERK1/2 pathway by RAFi, MEKi, ERKi or by expression of peptides or proteins (e.g. iDab6). We anticipate that the same system, modified as a survival screen, could be used to screen CRISPR, RNAi, protein or peptide-based libraries (https://data.epo.org/gpi/EP3218512B1-A-METHOD-OF-SCREENING-FOR-MODULATION-OF-CELL-SIGNALLING-PATHWAYS). These cell lines could be used to screen for novel inhibitors, to identify novel druggable space and to identify novel regulators that may represent new drug targets for the KRAS–RAF–MEK1/2–ERK1/2 pathway.

## Materials and methods

### Materials

Dulbecco's Modified Eagle's Medium (DMEM), FBS, Penicillin/Streptomycin and Glutamine were all purchased from Life Technologies. BIRB796 (S1574), CB1954 (S7829), PLX-4072 (S1267), Vemurafenib (S1267) and ZSTK474 (S1072) were purchased from Selleckchem; Trametinib (HY-10999) was from Insight Biotechnologies; JNK inhibitor VIII (CAS 894804-07-0) was from Calbiochem; SCH772984 (S7101) was from Merck. Bovine serum albumin (BSA), DMSO and Doxycycline were purchased from Sigma. Vectashield (H-1200-10) was purchased from VectorLabs. The following antibodies were all purchased from Cell Signalling Technologies: p-S473 AKT (4060), Akt1 (2967), c-Jun (9165), p-T202/Y204 ERK1/2 (4370), ERK1/2 (9107), GFP (2955), p-S217/S221 MEK1/2 (9154), MEK1/2 (4694), p-T334 MAPKAPK2 (8753), MAPKAPK2 (3042). BRAF (3967S) was purchased from Santa Cruz. Tubulin (T9026) and β-actin (A5441) were purchased from Sigma and KRAS (12063-1-AP) was from ProteinTech. Horseradish peroxidase-conjugated secondary antibodies were from Bio-Rad and the enhanced chemiluminescence (ECL) system from GE Healthcare was used for detection. The Dylight™680 and Dylight™800 conjugated secondary antibodies were from Cell Signalling Technology.

### Cell culture and reagents

Cell lines were grown in DMEM media supplemented with 10% (v/v) fetal bovine serum, penicillin (100 U/ml), streptomycin (100 mg/ml) and 2 mM glutamine (Life Sciences). Cells were maintained in a humidified incubator at 37°C and 5% (v/v) CO_2_. For inducible expression of oncogenes we used the the Flp-In TRex system from Invitrogen (ThermoFisher Scientific Cat No K650001). Parental Flp-In TRex tetracycline trans-activator HeLa cells (FRT/TO HeLa) (kind gifts from Stephen Taylor, University of Manchester) were used to generate doxycycline-inducible cell lines as described previously [16]. To generate HeLa cells with doxycycline-inducible BRAF^V600E^, *BRAF^1799T>A^* (encoding BRAF^V600E^) was PCR amplfii ed from pBabe-Puro-BRAF-V600E (Plasmid #15269, Addgene), cloned into pcDNA5/FRT/TO vector (Invitrogen) and confirmed by sequencing. Vectors were then cotransfected into (FRT/TO HeLa) with the Flp recombinase encoding plasmid pOG44 as described previously [16]. Stable integrants were selected with 4 μg/ml blasticidin (Invivogen) and 200 μg/ml hygromycin (Roche). Transgene expression was induced with 100 ng/ml Doxycycline (Sigma). A HeLa KRAS^G12D^ cell line was previously described [17]; a similar HeLa KRAS^G12V^ cell line was a kind gift from Amy Emery and Ashok R. Venkitaraman and was generated in the same manner as the HeLa BRAF^V600E^ cells. The HeLa EmGFP reporter cell lines were generated ‘in House’. Initially we tested a series of promoter deletion constructs (FL [−778 to +280], SRE 1–5 [−426 to+12] and SRE 1-2 [−113 to +12] numbered according to transcriptional start) which contained different numbers of SREs in front of the of the reporter gene, EmGFP. To generate stable reporter cell lines the FL EGR1 promoter region and 5′UTR were subcloned from pDRIVE-EGR1 (Invivogen) into pcDNA3.1 Zeo upstream of EmGFP. Both HeLa KRAS^G12V^ and BRAF^V600E^ heterogenous populations were then transfected with the FL EGR1:EmGFP reporter construct (FL-EGR) and single cell clones were selected in 50 µg/ml Zeocin. The HeLa NTR cells were generated by lentiviral transduction of a EGR1:NsfA–EGFP (encoding a NsfA–EGFP fusion) construct into both HeLa KRAS^G12V^ and BRAF^V600E^ and single cell clones were generated and screened.

### Transfection

Cells were cultured to ∼60% confluency. Constructs were then transfected using JetPrime polyplus transfection reagent or lipofectamine 3000 according to manufacturer's instructions. The iDab6 cDNA from pGC-IRES-iDab6 [22], a kind gift from Prof Terry Rabbitts, was cloned into a pcDNA3.1 vector with a V5 tag.

### SDS–PAGE and western blotting

Cells were lysed in TG lysis buffer and protein quantification performed by Bradford assay (Bio-Rad). Equal amounts of protein were then boiled with Laemelli sample buffer and fractionated by SDS–PAGE as described previously [34]. For detection by ECL, membranes were probed with HRP-conjugated secondary antibodies. Detection and quantification of blots after incubation with fluorescent secondaries (Cell Signalling Technologies) was performed using the LiCOR Odyssey system [35].

### High content microscopy

Cells were cultured in CellCarrier-96 plates (PerkinElmer). After the appropriate length of treatment with Dox ± inhibitors, cells were fixed with 4% formaldehyde/PBS (v/v) and permeabilised with 0.2% Triton X-100 for 10 min. For analysis of GFP intensity, cells were stained with DAPI at a concentration of 1 µg/ml for 10 min. For detection of p-ERK1/2, p-MEK1/2, total ERK1/2 or total MEK1/2, cells were blocked for 1 h with 2.5% goat serum (v/v) in 2% BSA/PBS (v/v) at room temperature. Cells were then incubated with primary antibody diluted in 2% BSA/PBS overnight at 4°C. Background control wells were treated with 2% BSA/PBS, without the addition of primary antibody. Cells were washed three times with PBS and then incubated with Alexa Fluor secondary antibodies (1:500) and DAPI (1 µg/ml) in 2% BSA/PBS for 1 h. Cells were washed with PBS before imaging. Cells were imaged using an INCELL Analyzer 6000 Microscope (GE Healthcare), imaging six fields per well. Image analysis to determine the intensity of GFP or fluorescent signal was performed using INCELL Analyzer software.

### Confocal microscopy

Cells were seeded in 12-well plates containing coverslips. Cells were incubated for 24 h and then treated as described in the figure legend. Cells were fixed with 4% paraformaldehyde/PBS (v/v) for 15 min at room temperature and washed in PBS. Coverslips were washed in water (MiliQ) and mounted on glass slides with Vectashield mounting media containing DAPI and imaged using a Leica Stellaris 8 microscope with a 60× objective lens. Image analysis was performed using ImageJ software.

### Cell viability

HeLa NTR5 or NTR7 cells (clones harbouring EGR1:NsfA) were cultured in six well plates. After treatment with Dox cells were fixed in Methanol/acetic acid (3:1 v:v) for 10 min then stained in 0.1% crystal violet solu-tion in PBS (100 mg/100 ml, w/v) for 10 min. Cells were solubilised in 10% acetic acid and absorbance was read at 590 nm on a plate reader (Pherastar).

### Data reproducibility

All individual experiments described have been repeated at least three times, and many have been repeated between four and six times. Initial observations were made by Rebecca Gilley, Kate Stuart and Megan Cassidy. These were reproduced independently and then expanded on, with mechanistic analysis, by Laura Weatherdon who generated the bulk of the data and performed analaysis, quantification and statistical analysis. So key results have been repeated independently by up to four experimentalists.

## Data Availability

All relevant data are contained within the main article and its supplementary files.

## Abbreviations

BSA: bovine serum albumin
CRE: cAMP response element
DMEM: Dulbecco's Modified Eagle's Medium
ECL: enhanced chemiluminescence
EmGFP: Emerald green fluorescent protein
FBS: foetal bovine serum
FL: full length
GDEPT: gene-directed enzyme prodrug therapy
SRE: serum response element
TRE: tetracycline response element
