# Supplementary material for: Reporter cell lines to screen for inhibitors or regulators of the KRAS-RAF-MEK1/2-ERK1/2 pathway
Source: Biochem J. 2024 Mar 7;481(6):405–22. doi: 10.1042/BCJ20240015 (PMC11088904; doi:10.1042/BCJ20240015)
Supplement: Supplementary Material 1 [file BCJ-481-405-s1.pdf]

Supplementary Figure legends

Supplementary Figure 1. The Full length promoter and 5'UTR are required for maximal induction of the reporter gene. (A) Schematic showing the deletion constructs tested for minimal EGR1 promoter activity. The promoter region and 5' UTR of EGR1 (-778 to +280) inserted upstream of the EmGFP cDNA. The promoter consists of five critical ERK1/2 responsive serum-response elements (SREs), as well as two cAMP-responsive elements (CREs) and two SP1 elements. To delineate the minimal required upstream elements deletion constructs were made containing either all 5 serum responsive elements (SRE1-5, -426 to +12) or just the first 2 that have previously been described as minimally essential (SRE1-2, -113 to +12). **(B)** Reporter constructs containing the above upstream regions were transfected into cells and these were then treated with 100 ng/ml doxycycline (Dox) or DMSO for 24 hours, fixed and stained with DAPI before being imaged on a high content microscope. Results were analysed to determine the percentage of GFP positive cells within the population.

Supplementary Figure 2. Inhibitors of PI3K, p38 MAPK or JNK do not inhibit KRAS^{G12V} or BRAF^{V600E} dependent ERK1/2 signalling or EmGFP expression. (A) HeLa cells containing the EGR1:EmGFP reporter construct and expressing doxycycline-inducible KRAS^{G12V} (KR1) or BRAF^{V600E} (BR5) were treated with 100nM Trametinib (Tram), 200nM ZSTK474 (ZSTK), 50 nM BIRB796 (BIRB), or 5µM JNK inhibitor VIII (JNK) for 24hrs. All cells were then treated with 500 ng/ml Anisomycin for 1 hour before lysis, whole cell lysates were fractionated by SDS-PAGE and blotted with the indicated antibodies. Results are representative of three independent experiments. **(B)** KR1 and BR5 cells were treated with inhibitors as above and also treated with 100 ng/ml Doxycycline (Dox) or DMSO, whole cell lysates were fractionated by SDS-PAGE and blotted with the indicated antibodies

A

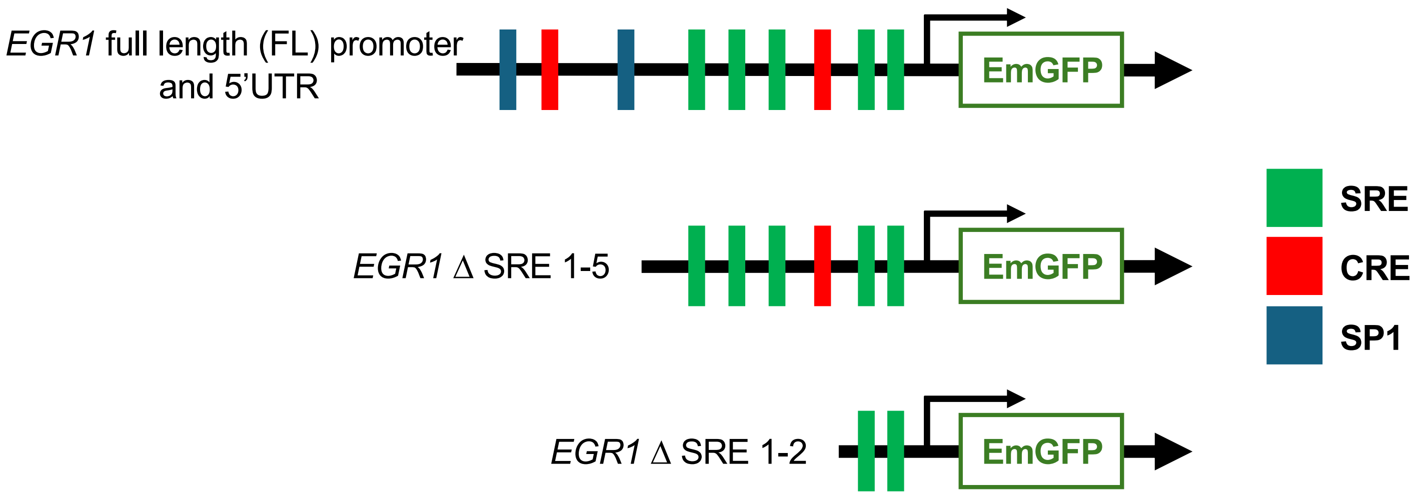
EGR1 full length (FL) promoter
and 5'UTR
EmGFP
EGR1 Δ SRE 1-5
EmGFP
EGR1 Δ SRE 1-2
EmGFP
SRE
CRE
SP1

B

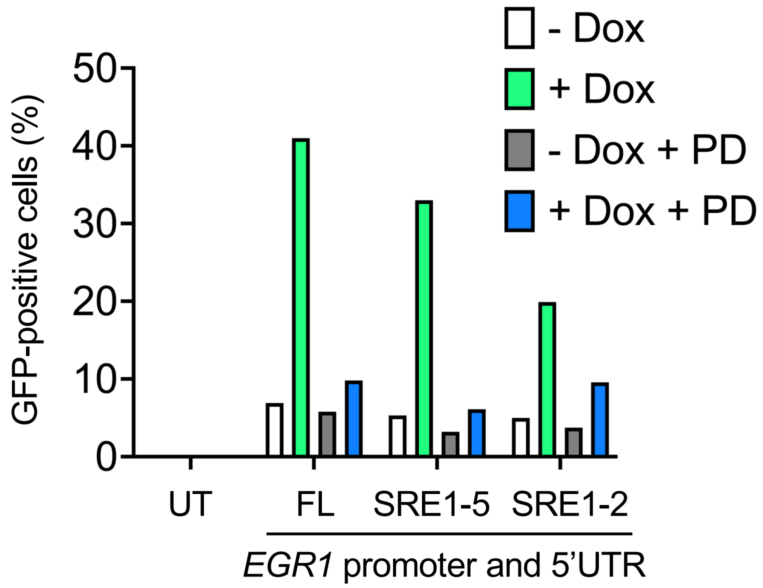
- Dox
+ Dox
- Dox + PD
+ Dox + PD
GFP-positive cells (%)
50
40
30
20
10
0
UT
FL
SRE1-5
SRE1-2
EGR1 promoter and 5'UTR

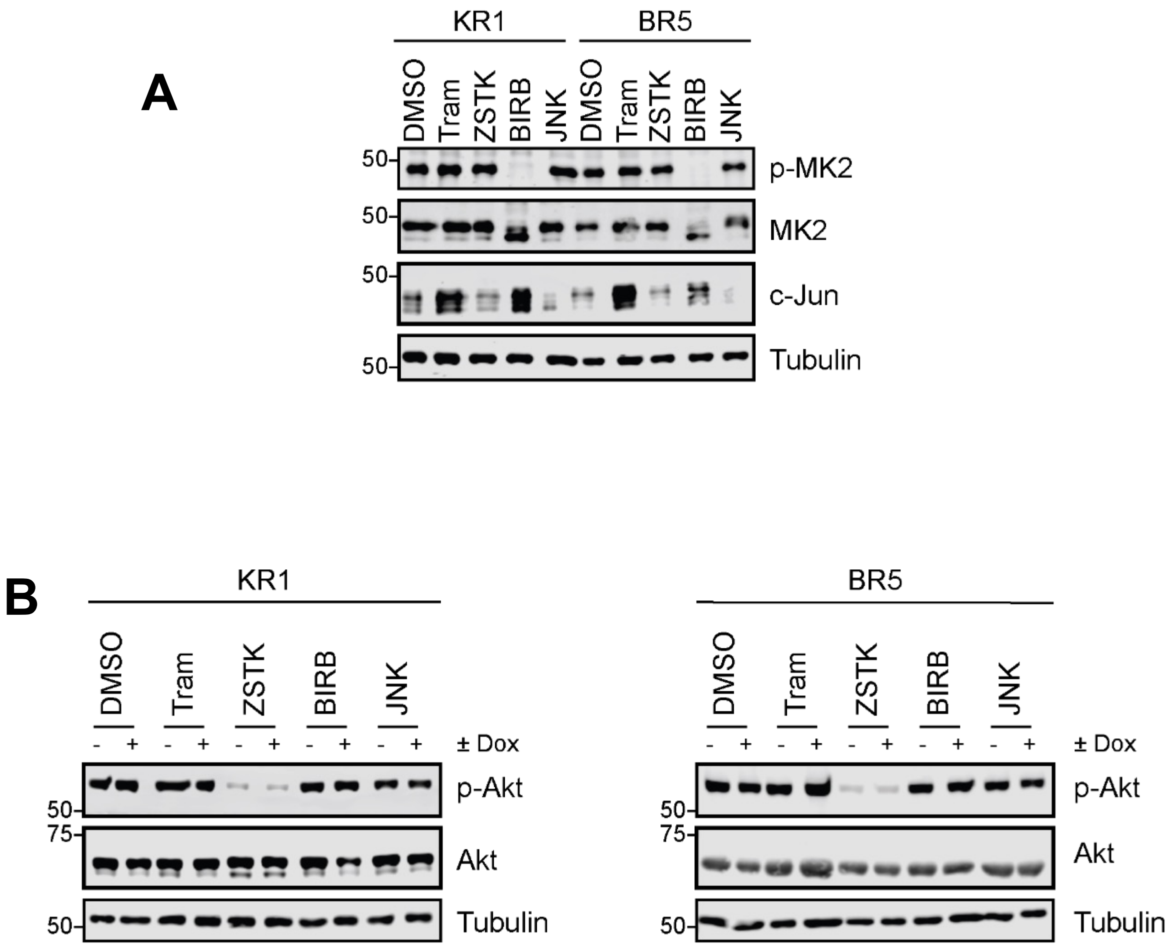
A
KR1
BR5
DMSO
Tram
ZSTK
BIRB
JNK
DMSO
Tram
ZSTK
BIRB
JNK
50
p-MK2
50
MK2
50
c-Jun
50
Tubulin
B
KR1
DMSO
Tram
ZSTK
BIRB
JNK
- + - + - + - + - +
± Dox
p-Akt
50
75
Akt
50
Tubulin
BR5
DMSO
Tram
ZSTK
BIRB
JNK
- + - + - + - + - +
± Dox
p-Akt
50
75
Akt
50
Tubulin
